# Chloroform in Puerperal Convulsions

**Published:** 1850-11

**Authors:** 


*Chloroform in Puerperal Convulsions.*—Dr. Keith gives the following particulars of a case of puerperal convulsions treated by chloroform. A lady, about the end of the seventh month of her first pregnancy, was suddenly seized with a convulsive fit, after suffering more or less for some days from headache, and slight feverish symptoms. The first fit occurred three hours before Dr. K. saw her, and in the interval she had three others. She had been bled, and the bowels had been freely opened. The fits ceased for a time, but after four hours they again recurred, and four followed each other in the course of less than an hour, without any return to sensibility during the intervals. The bleeding was repeated very copiously, but seemed to have no effect in stopping the fits, or even in rendering them more moderate. Just as the fourth fit was coming on, the chloroform was administered. It had an immediate effect in restraining the convulsive efforts, and she soon became perfectly quiet. Dr. K. had ascertained, on first seeing the patient, that the os uteri was quite shut, and there had been no sign of labor coming on up to this period. Now, however, from the motions and expression of countenance, while she was still under the chloroform, he was led again to examine as to the state of the uterus, and found that regular contractions had come on. The action of chloroform was kept up, the labor went on steadily, and in six hours she was delivered of a still-born fœtus, without any recurrence of the convulsions. She slept for nearly an hour after the termination of the labor, when she awoke in a confused state, but perfectly rational, and almost free from headache. She made a most favorable recovery; indeed, she had not a single bad symptom from the time that the chloroform was administered. The urine was highly albuminous, becoming almost solid on exposure to heat. The coagulability gradually went off, and after a fortnight there was scarcely a trace of it.—*Monthly Jour. of Med.*, Aug., 1850.

